# A sorghum *gigantea* mutant attenuates florigen gene expression and delays flowering time

**DOI:** 10.1002/pld3.281

**Published:** 2020-11-13

**Authors:** S. M. Abdul‐Awal, Junping Chen, Zhanguo Xin, Frank G. Harmon

**Affiliations:** ^1^ Plant Gene Expression Center USDA‐ARS Albany CA USA; ^2^ Department of Plant & Microbial Biology University of California Berkeley CA USA; ^3^ Biotechnology & Genetic Engineering Discipline Khulna University Khulna Bangladesh; ^4^ Plant Stress and Germplasm Development Unit USDA‐ARS Lubbock TX USA

**Keywords:** Blue light signaling, FKF1, florigen, flowering time, gene expression, GIGANTEA, photoperiodic flowering, *Sorghum bicolor*

## Abstract

*GIGANTEA* (*GI*) is a conserved plant‐specific gene that modulates a range of environmental responses in multiple plant species, including playing a key role in photoperiodic regulation of flowering time. The C4 grass *Sorghum bicolor* is an important grain and subsistence crop, animal forage, and cellulosic biofuel feedstock that is tolerant of abiotic stresses and marginal soils. To understand sorghum flowering time regulatory networks, we characterized the *sbgi‐ems1* nonsense mutant allele of the sorghum *GIGANTEA* (*SbGI*) gene from a sequenced M4 EMS‐mutagenized BTx623 population. *sbgi‐ems1* plants flowered later than wild type siblings under both long‐day or short‐day photoperiods. Delayed flowering in *sbgi‐ems1* plants accompanied an increase in node number, indicating an extended vegetative growth phase prior to flowering. *sbgi‐ems1* plants had reduced expression of floral activator genes *SbCO* and *SbEHD1* and downstream FT‐like florigen genes *SbFT*, *SbCN8*, and *SbCN12*. Therefore, *SbGI* plays a role in regulating *SbCO* and *SbEHD1* expression that serves to accelerate flowering. SbGI protein physically interacts with the sorghum FLAVIN‐BINDING, KELCH REPEAT, F‐BOX1‐like (SbFFL) protein, a conserved flowering‐associated blue light photoreceptor, and the SbGI‐SbFFL interaction is stimulated by blue light. This work demonstrates that SbGI is an activator of sorghum flowering time upstream of florigen genes under short‐ and long‐day photoperiods, likely in association with the activity of the blue light photoreceptor SbFFL.

**Significance Statement:**

This study elucidates molecular details of flowering time networks for the adaptable C4 cereal crop *Sorghum bicolor*, including demonstration of a role for blue light sensing in sorghum GIGANTEA activity. This work validates the utility of a large publicly available sequenced EMS‐mutagenized sorghum population to determine gene function.

## 1 INTRODUCTION

*GIGANTEA* (*GI*) is a gene identified in early genetic screens for delayed flowering mutants in *Arabidopsis thaliana* (Koornneef et al., 1991; Redei, 1962). *GI* participates in flowering time control, the circadian clock, and a wide range of other physiological activities (Mishra & Panigrahi, 2015). *Arabidopsis GI* stimulates flowering by promoting *FLOWERING LOCUS T* (*FT*) expression under long day (LD) photoperiods through post‐transcriptional inactivation of *CONSTANS* (*CO*) transcriptional repressors (Park et al., 1999; Sawa & Kay, 2011; Sawa et al., 2007; Suarez‐Lopez et al., 2001). In this capacity, GI interacts with the blue light photoreceptor FLAVIN‐BINDING, KELCH REPEAT, F‐BOX1 (FKF1) as part of the E3 ubiquitin ligase that targets a family of *CO* transcriptional repressors for degradation by the 26S proteasome system (Sawa et al., 2007). GI is also implicated in direct transcriptional regulation of *FT* (Sawa & Kay, 2011). The CO‐FT regulatory module is a highly conserved point of integration for photoperiodic flowering signals (Song et al., 2015). CO is a B‐box CCT domain transcription factor and its primary role is control of *FT* expression (Griffiths et al., 2003; Putterill et al., 1995). FT is a PEBP‐family protein (Danilevskaya et al., 2008; Turck et al., 2008), which acts as florigen, a mobile signal transmitting the flowering signal from leaves to the shoot apical meristem (SAM; Pennazio, 2004). Leaf expressed FT‐like proteins in *Arabidopsis*, rice, tomato, and cucurbits trigger the SAM to transition from vegetative to floral developmental programs (Jaeger & Wigge, 2007; Lifschitz et al., 2006; Lin et al., 2007; Notaguchi et al., 2008; Tamaki et al., 2007).

*GI* is also an important component of photoperiodic flowering time networks in grasses. Rice and maize *gi* mutants alter flowering time behavior. The rice *osgi‐1* mutant allele delays flowering under short day (SD) but not LD photoperiods under greenhouse conditions, and only slightly delays flowering in the field (Izawa et al., 2011). *OsGI* is important for blue light‐promoted induction of rice *Early heading date 1* (*OsEHD1*) expression as part of the mechanism for critical SD day‐length recognition (Itoh et al., 2010). *OsEHD1* encodes a B‐type response regulator that promotes expression of the FT homolog *OsHd3a* under SD separate from the CO homolog *OsHd1* (Doi et al., 2004; Itoh et al., 2010; Zhao et al., 2015). Maize has two paralogous *GI* genes, *GIGANTEA1 (GI1)* and *GIGANTEA2* (Mendoza et al., 2012; Miller et al., 2008). *gi1* mutants flower earlier in LD, but not SD, and have elevated expression of *FT* homolog *Zea mays CENTRORADIALIS 8 (ZCN8)* and *CO* homolog *CONSTANS OF Zea mays1 (CONZ1)*, indicating that *GI1* is an upstream repressor in LD (Bendix et al., 2013). The function of maize GI1 is sufficiently conserved that it complements late flowering of an *Arabidopsis gi* knockout mutant (Bendix, 2015).

Sorghum is a C4 grass native to Africa that is a key grain and subsistence crop, an animal forage, and a promising cellulosic biofuel feedstock. Sorghum is highly stress tolerant, maintaining productivity in marginal soils and under arid conditions. Sorghum is originally a short day (SD) flowering plant in which long dark periods, and correspondingly short days, above a critical threshold promote flowering (Craufurd et al., 1999; Quinby, 1974). Selection of so called *Maturity (Ma)* loci, which reduce the SD requirement for promotion of flowering, has allowed expansion of sorghum cultivation to more northern latitudes (Quinby, 1974). Of these, the *Ma1* locus has the largest impact on sorghum flowering time. Inactive *ma1* alleles confer early flowering in LD conditions and played an important role in early domestication of sorghum (Quinby, 1967).

Murphy et al. demonstrated that differentially functional sorghum *PSEUDORESPONSE REGULATOR 37* (*SbPRR37*) alleles underlie the *Ma1* locus (Murphy et al., 2011). *SbPRR37* encodes a member of a family of transcriptional repressors originally discovered as core circadian clock genes in Arabidopsis (Farre & Liu, 2013), but *SbPRR37* has no contribution to circadian clock function (Murphy et al., 2011). Under LD conditions, *SbPRR37* inhibits flowering by repressing expression of flowering activators *SbEhd1* and *SbCO* to ultimately suppress expression of *SbFT*, *SbCN8*, and *SbCN12* (Murphy et al., 2011).

Like its rice and Arabidopsis counterparts, sorghum *CONSTANS* (*SbCO*) acts upstream to promote expression of *SbEhd1* and several florigen‐related genes in both LD and SD photoperiods (Yang et al., 2014). Of the thirteen PEBP‐family genes in sorghum, sorghum *CENTRORADIALIS 8* (*SbCN8*) is the co‐linear ortholog of maize *ZCN8* and *SbFT* is the co‐linear ortholog of rice *Hd3a* (Murphy et al., 2011). An additional PEBP‐family gene orthologous between maize and sorghum is *SbCN12* (Murphy et al., 2011; Yang et al., 2014). Both *SbCN8* and *SbCN12* possess florigen activity when overexpressed in Arabidopsis (Wolabu et al., 2016). Collectively, *SbFT*, *SbCN8*, *SbCN12* are regulated by *SbCO* and *SbEhd1* (Murphy et al., 2014; Yang et al., 2014), consistent with this set of genes acting as the CO‐FT module in sorghum.

The contribution of the sorghum *GI* (*SbGI*) gene to regulation of sorghum flowering is not well‐characterized. A comparative genomic study of 219 African sorghum accessions identified single nucleotide polymorphisms (SNPs) at *SbGI* significantly associated with photoperiod sensitivity (Bhosale et al., 2012). Two associated SNPs caused non‐synonymous amino acid changes and a third represented a frameshift mutation. Like all known *GI* genes, *SbGI* expression has a diel rhythm where peak expression occurs 9–12 hr after dawn (Lai et al., 2020; Murphy et al., 2011). Diurnal expression of *SbGI* is close to that of maize *GI1* and expression of both *SbGI* and *GI1* is substantially higher than *GI2* (Lai et al., 2020). *SbPRR37* does not contribute substantially to regulation of *SbGI* (Murphy et al., 2011).

Here we characterize a mutant allele in the *SbGI* gene, *sbgi‐ems1*, from a sequenced M4 EMS‐mutagenized population (Jiao et al., 2016). Plants carrying this nonsense mutation, which truncates GI protein by two thirds, exhibit delays in flowering under LD and SD photoperiod conditions. Delayed flowering in *sbgi‐ems1* under LD accompanies an increase in node number, indicating an extended vegetative growth phase prior to flowering. Mutant plants had low expression of *SbCN8* and *SbCN12* under LD and SD photoperiods. Also, our observations indicate that *SbGI* promotes expression of the *SbCO* under both LD and SD photoperiods, but contributes to peak *SbEHD1* expression mainly under SD. Testing of the molecular activity of SbGI showed that it physically interacts with the sorghum FKF1‐like (SbFFL) protein, a potential flowering‐associated blue light photoreceptor, and the SbGI‐SbFFL interaction is stimulated by blue light.

## 2 MATERIALS AND METHODS

### 2.1 Plant stocks and environmental conditions

All sorghum lines are the BTx623/ATx623 genetic background. The ARS223 line is from a collection of 256 whole genome sequenced M4 EMS‐mutagenized sorghums lines described previously (Jiao et al., 2016). Plants were screened for the *sbgi‐ems1* mutation in *SbGI* by Derived Cleaved Amplified Polymorphic Sequences PCR with the primers in Table S1. The PCR fragment amplified from the *sbgi‐ems1* locus is resistant to the XcmI restriction enzyme (New England Biolabs, www.neb.com) and the product from WT *SbGI* locus is cleaved by this enzyme. One plant carrying the *sbgi‐ems1* allele from the M4 ARS223 population was used as pollen donor for a cross to a male sterile ATx623 panicle. Progeny of this cross were used for subsequent experiments and backcrossing.

LD conditions in the greenhouse were 16‐hr days and 8‐hr nights. Natural sunlight was supplemented with LumiGrow Pro325 LEDs (www.lumigrow.com) set at maximum intensity for all channels. SD conditions were in the greenhouse under natural sunlight only. Daytime temperature was set to 26°C and nighttime temperature was set to 20°C. Seedlings for growth measurements and gene expression were sown in 4‐inch peat pots filled with SuperSoil (The Scotts Company, www.scotts.com), supplemented with a ½ teaspoon of 14‐14‐14 N‐P‐K slow release fertilizer. Plants for flowering experiments were started in the same fashion then transplanted when seedlings reached the 3‐leaf stage (10 days‐old) to 13‐liter pots filled with corn soil (composed of aged wood fines, green waste compost, fir bark, grape compost, rice hulls, chicken manure, red lava, and sandy loam mixed by American Soil and Stone, Richmond, CA). Greenhouse plants were watered twice daily and received 20‐20‐20 N‐P‐K fertilizer once a week after being transplanted to 13‐liter pots. Field grown plants for year 2018 (late May to September) were maintained in rows at Oxford tract on the University of California, Berkeley campus and watered to soil saturation once weekly by drip irrigation, and for year 2019 (early June to August) were maintained in rows at the Vegetable Crop Field Station on the University of California Davis campus and watered to soil saturation once weekly by drip irrigation. For each trial, field grown plants were started from seed directly or transplanted as 4–5 leaf individuals (2 weeks‐old) started is peat pots as above.

### 2.2 Assessment of flowering time

Plants were individually scored for the number of days from sowing to reach boot stage and flowering, while field grown plants were scored for boot stage only, due poor pollen shed and stigma exertion at the UC Berkeley Oxford tract. Boot stage was scored as the first day when the entire flag leaf collar was visible in the leaf whorl. Flowering stage was scored as the first day of anthesis for fertile plants or stigma exertion for male sterile plants.

### 2.3 Analysis of gene expression by qPCR

Plants at the fifth to sixth leaf stage grown under LD greenhouse conditions were transferred to a growth chamber set to either LD (16 hr light; 8 hr darkness) or SD (10 hr light; 14 hr darkness) with daytime temperature of 28°C and nighttime temperature of 23°C until all plants reached the fifth to seventh leaf stage. These plants were sampled at 0, 6, 12, and 18 hr after lights came on. Leaf samples were taken by cutting directly across the 6th leaf ligule with scissors. Three or two biological replicates were collected for each genotype at each time point. A biological replicate consisted of pooled tissue from three individuals of the same genotype. Leaf samples were flash frozen in liquid nitrogen.

After tissue was ground under liquid nitrogen, total RNA was extracted with TRIzol Reagent (ThermoFisher Scientific, www.thermofisher.com) according to the manufacturer's recommendations. Total RNA (3.5 µg) for each sample was treated with dsDNase (ThermoFisher Scientific, www.thermofisher.com) to remove contaminating genomic DNA and was used as a template for cDNA synthesis with the Maxima H Minus First Strand cDNA synthesis Kit (ThermoFisher Scientific, www.thermofisher.com) according to the manufacturer's recommendations. cDNA diluted in half with water served as template for two technical replicate real‐time quantitative PCR (qPCR) reactions composed and performed as previously described (Bendix et al., 2013). qPCR reactions for normalization employed PCR primers for 18S RNAs (Table S1) and cDNA diluted an additional 1:2000 in water. C_q_ values were calculated with the regression function for each primer set in the Bio‐Rad CFX Manager Software (Bio‐Rad, www.bio‐rad.com) and values of relative transcript levels were calculated as 2^(*C_q_^18S^*‐ *C_q_^experimental^*). For each replicate, relative expression at a time point was calculated by dividing the relative transcript value by the average of all relative transcript values in that replicate. Standard deviation was calculated from all relative expression values for that time point.

### 2.4 Protein interaction analysis by yeast two‐hybrid

The coding sequences of SbGI and SbFFL were amplified by PCR with Q5 High Fidelity Polymerase (New England Biolabs, www.neb.com) from cDNA with the primers in Table S1. PCR products were cloned into pENTR/D‐TOPO vector (ThermoFisher Scientific, www.thermofisher.com) and sequences confirmed by Sanger sequencing. *SbFFL* and *SbGI* cDNA sequences were subcloned into bait vector pGBKT7‐Rec and the prey plasmid pGADT7‐Rec, respectively, with LR Clonase II (ThermoFisher Scientific, www.thermofisher.com). Bait and prey plasmids were transformed into Y2H Gold yeast cells according to the manufacturer's recommendations (Takara Bio, www.takarabio.com). For interaction tests, two individual transformants for each plasmid combination were grown at 30°C in liquid Synthetic Dropout (SD) media lacking amino acids Leu (L) and Trp (W) supplemented with 50 μg/mL Kanamycin (SD‐T‐W) to an absorbance of 600 nm (A_600_) = 1.0, then samples were prepared corresponding to cell densities with A_600_ = 4, 2, 1, 0.5, 0.1, and 0.01. 10 μL of each sample was spotted on SD‐L‐W plates with or without 200 ng/ml of the antibiotic Aureobasidin A (Takara Bio USA, www.takarabio.com). Interaction between bait and prey proteins confers resistance to Aureobasidin A. After drying, plates were sealed with Micropore Paper Tape (3M, www.3m.com) and placed under continuous blue light provided by blue LEDs at 25–30 μmol/m^2^ s or continuous darkness at 30**°**C in a Percival LED‐30 growth chamber. Digital images of plates were taken after 3, 5, and 7 days to monitor yeast growth.

### 2.5 Transient infiltration of *Nicotiana benthamiana* and bimolecular fluorescence complementation

The SbGI and SbFFL coding sequences in pENTR/D‐TOPO were subcloned by LR Clonase II (ThermoFisher Scientific, www.thermofisher.com) into pB7WGYc2 and pB7WGYn2 respectively. Empty pB7WGYc2 and pB7WGYn2 constructs were made by LR Clonase II reactions with water in place of the pENTR/D‐TOPO vector. Each construct was transformed into *Agrobacterium tumefaciens* strain GV3101 via electroporation. The resultant *Agrobacterium* strains and the strain GV2260, carrying the P19 silencing suppressor vector, were pressure infiltrated into *N. benthamiana* leaves. Overnight *Agrobacterium* cultures in LB broth supplemented with appropriate antibiotics were pelleted and resuspended in 10 ml of induction medium (50 mM MES pH 5.6, 0.5% (w/v) glucose, 1.7 mM NaH2PO4, 20 mM NH4Cl, 1.2 mM MgSO4, 2 mM KCl, 17 μM FeSO4, 70 μM CaCl2, and 200 μM acetosyringone). Cultures were incubated at 30°C for 6 hr, the cells were pelleted and resuspended in 10 mM MES (pH 5.6) in presence of 200 μM acetosyringone at an A_600_ = 1.0. The cultures containing each plasmid were mixed in equal volumes to a final A_600_ = 0.25 per construct. P19 silencing suppressor culture was at this same cell density in all infiltrations. The underside of the leaves of 5‐ to 7‐week‐old *N. benthamiana* plants were infiltrated by hand with a needleless syringe. Infiltrated plants were subsequently transferred to continuous blue light provided by blue LEDs at 25–30 μmol/m^2^ s or continuous darkness in a Percival LED‐30 growth chamber at 22**°**C for 24–48 hr prior to imaging by confocal microscopy.

### 2.6 Confocal microscopy

Small sections (0.5 cm^2^) of infiltrated *N. benthamiana* leaves were infiltrated with water and were mounted on microscope slides. Samples were imaged using a Leica SP8 confocal laser‐scanning microscope equipped with a 20x water‐immersion objective. The 514 nm argon laser line was used to excite YFP, and florescence was observed using the specific emission window of 520–600 nm. The laser power (Argon intensity 25%), gain (1,050), zoom (zoom factor 1), and average settings (Format 1024x1024; Speed 200; line average 2; line accuracy 1; frame average 2; frame accuracy 1) were kept consistent over the same image series to allow fluorescence intensity comparison across samples. Images were processed using the Leica Application Suite X software package.

## 3 RESULTS

### 3.1 *gi‐ems1* is a nonsense EMS mutation in *SbGI*

A single *GI* gene is present in the sorghum genome on the short arm of chromosome 3 (position 3:3,821,973‐3,830,666; Sobic.003G040900; SORBI_3003G040900; Lai et al., 2020). Publicly available RNAseq analysis shows that *SbGI* is widely expressed in juvenile and adult tissues, with expression higher in leaf, shoot, and root‐related tissues compared to flower‐ and seed‐associated tissues (Figure S1a; Davidson et al., 2012; Olson et al., 2014; Makita et al., 2015).

The SbGI protein is 68% identical to the *Arabidopsis* GI protein (Data S1). SbGI shares 96.47% and 96.21% amino acid identity with maize orthologs GI1 and GI2, respectively (Data S1). Interestingly, 80.5% of the variant residues are shared between SbGI and only one of the maize orthologs, instead of residues identical between GI1 and GI2 (Data S1). Indeed, 43% of the total variants are only shared between SbGI and GI1, while 37.5% are only shared between SbGI and GI2.

**FIGURE 1:**
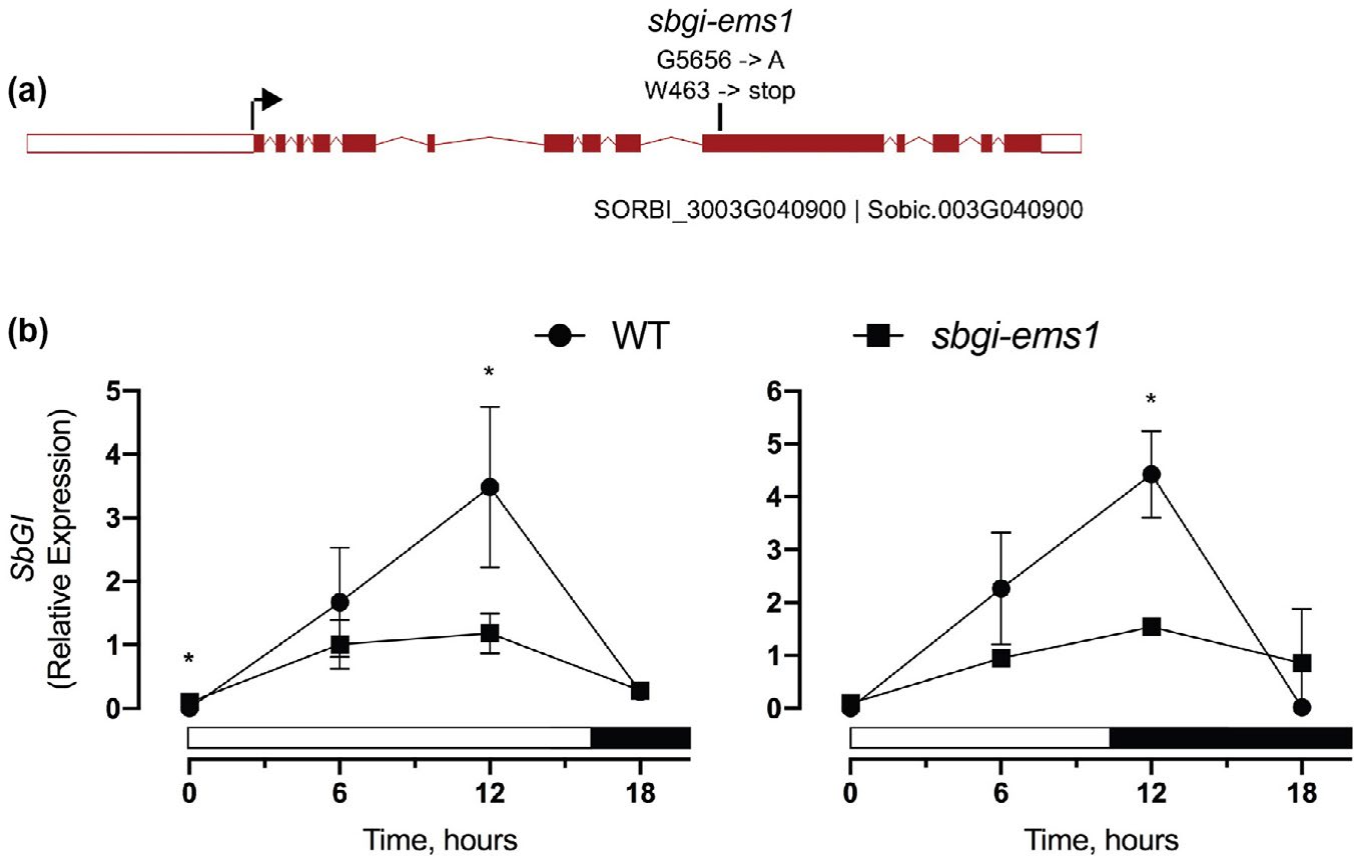
The *sbgi‐ems1* nonsensus mutation causes reduced *SbGI* expression. (a) Diagram of *SbGI* gene (Sobic.003G040900; SORBI_3003G040900) where boxes indicate exons and lines introns. Red coloring indicates coding sequence with the arrow at start codon and white regions indicating 5′‐ and 3′‐UTRs. Vertical line above indicates the position and nature of the *sbgi‐ems1* mutation. (b) Relative expression of *SbGI* in leaves of WT (circles) and *sbgi‐ems1* (squares) BC1F3 plants at the sixth leaf stage grown under LD (left) or SD (right) photoperiods. Time points are the average of three biological replicates and error bars are the standard deviation. X‐axis is the number of hours after dawn, white and black bars indicate light and dark periods, respectively. Statistical significance is indicated according to the two‐tailed Student's *t*‐test at *p* < .05 (*)

To evaluate the function of *SbGI*, we took advantage of an uncharacterized mutant allele in a collection of M4 EMS‐mutagenized BTx623 lines described previously (Jiao et al., 2016). The ARS223 line carries an EMS‐induced G to A mutation in *SbGI* at nucleotide position 5,656 (Figure 1a). This mutant allele, named here *sbgi‐ems1*, introduces a premature stop codon in place of a conserved tryptophan (W463*; Data S1). This allele truncates the normally 1,162 amino acid SbGI protein by nearly two thirds to a 462 amino acid protein. Individual plants carrying the *sbgi‐ems1* allele were identified in the original ARS223 material by PCR genotyping and the nature of the mutation was confirmed by sequencing. One carrier of the *sbgi‐ems1* allele was crossed to a male sterile ATx623 individual to complete backcross 1 (BC1) and subsequent backcrosses. Homozygous *sbgi‐ems1* plants (BC1F3) have reduced overall and peak expression of *SbGI* under LD photoperiods (Figure 1b), as is frequently observed for nonsense alleles. This reduction in gene expression and the nature of the *sbgi‐ems1* mutation together indicate that this allele causes significant disruption of *SbGI* activity.

### 3.2 *sbgi‐ems1* imparts delayed flowering under LD and SD photoperiods

**FIGURE 2:**
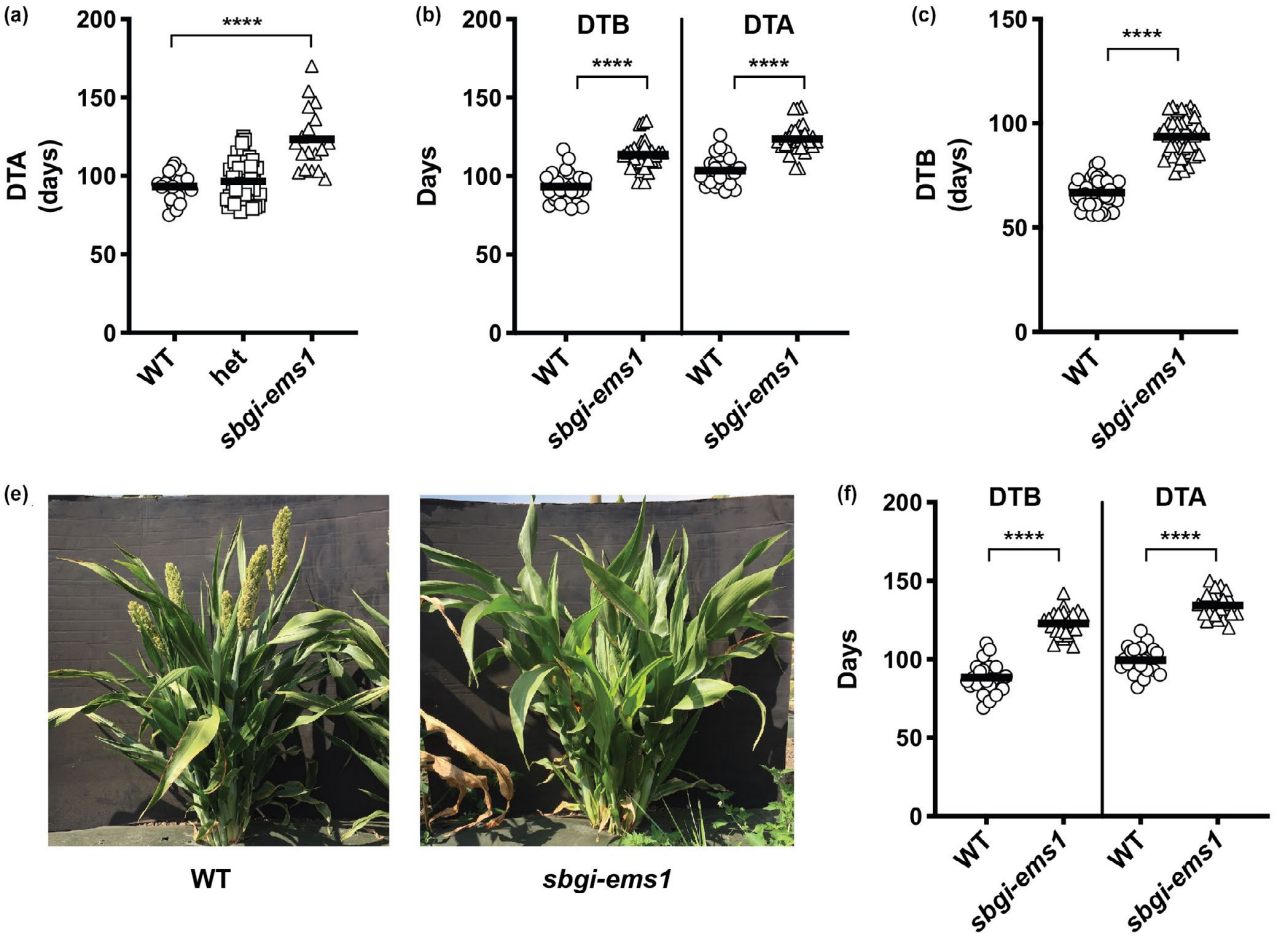
*sbgi‐ems1* mutants are late flowering. (a) Flowering time for BC1F2 population for WT (circles), heterozygous *sbgi‐ems1/SbGI* (squares), and *sbgi‐ems1* (triangles) plants grown under LD greenhouse conditions are determined as DTA). (b) Flowering time as DTA and DTB for BC1F3 WT (circles) and *sbgi‐ems1* (triangles) plants grown under LD greenhouse conditions. (c) DTB for BC1F3 *sbgi‐ems1* (triangles) and WT sibling (circles) plants grown under field conditions in Berkeley, CA during summer of 2018. (d) Pictures of representative 3 month‐old WT and *sbgi‐ems1* plants grown in the Berkeley field. (e) Flowering time as DTA and DTB for BC1F3 WT (circles) and *sbgi‐ems1* (triangles) plants grown under SD during the winter in the greenhouse. All measurements are shown from at least two separate trials, bar represents the average of measurements. Statistical significance is indicated according to a two‐tailed unpaired *t*‐tests with Welch's correction at p < .0001 (****)

Testing the flowering time of mutant and wild type (WT) plants grown under LD photoperiods (16 hr light; 8 hr dark) indicated that the *sbgi‐ems1* allele was associated with delayed flowering. Flowering time was determined in the greenhouse in four separate trials for a BC1F2 population segregating for *sbgi‐ems1* (total number of plants = 91). Flowering time was scored as days to anthesis for male fertile plants or days to the exertion of stigma for male sterile plants (collectively referred to here as Days To Anthesis or DTA). The timing of DTA was indistinguishable between plants genotyping as WT at *SbGI* (Figure S2a). The DTA for *sbgi‐ems1* homozygous plants averaged 30 days more than *sbgi1‐ems1* heterozygous and *SbGI* WT siblings (Figure 2a), while heterozygous *sbgi‐ems1* plants reached DTA an average of four days later than WT plants, a difference that is not statistically significant. The late flowering trait co‐segregated with the homozygous *sbgi‐ems1* genotype (Figure S2b). DTA was also greater by an average of 20 days for *sbgi‐ems1* mutant BC1F3 plants under LD conditions in two separate greenhouse trials (Figure 2b). Similarly, *sbgi‐ems1* plants from backcross 2 (BC2) and backcross 4 (BC4) showed a significant delay in flowering: BC2F3 and BC4F3 mutant plants reached anthesis later than WT siblings by an average of 31 and 50 days respectively (Figure S2c,d). These findings confirmed the link between the *sbgi‐ems1* mutation and delayed flowering time.

The days required to reach boot stage (Days To Boot or DTB), an indicator of flowering apparent prior to anthesis, was also delayed by the *sbgi‐ems1* allele. BC1F3 s*bgi‐ems1* plants reached boot stage an average of 20 days later than WT siblings (Figure 2b). In similar fashion, the DTB for *sbgi‐ems* BC2F3 and BC4F3 plants was an average of 29 and 51 days greater, respectively, than WT siblings (Figure S2c,d). These observations indicated that a delay in reaching boot stage was a major contributor to later flowering in *sbgi‐ems1*. Calculation of the time interval between DTB and DTA for all BC1 flowering time trials showed a statistically significant decrease of 1.5 days in the time it took *sbgi‐ems1* plants to proceed from boot stage to anthesis/stigma exertion (Figure S2e). Thus, the primary cause of the flowering delay in *sbgi‐ems1* was slower progression to boot stage.

The flowering time of *sbgi‐ems1* plants grown in the field during the summer also was later than their WT siblings in field trials conducted at two different locations in successive years. DTB was determined for BC1F3 *sbgi‐ems1* and WT sibling plants grown in Berkeley, CA during the summer of 2018 and in Davis, CA during the summer of 2019. In these two trials, the average DTB for *sbgi‐ems1* plants was greater than WT sibling plants by 28 (Berkeley) and 22 (Davis) days (Figure 2c,d; Figure S2f). These results show that *SbGI* is an important flowering time gene for sorghum that contributes to promotion of flowering under LD conditions.

Testing of flowering time under natural SD photoperiods indicated *SbGI* also plays a role in flowering under these conditions. DTB and DTA were measured for *sbgi‐ems1* and WT sibling plants in two trials where plants were gown in the greenhouse beginning in December of 2019 without supplemental lighting. Under these winter sunlight conditions, DTB and DTA both were increased in *sbgi‐ems1* plants by an average of 35 days relative to their WT siblings (Figure 2e). Thus, *SbGI* contributes to determination of flowering time under both LD and SD photoperiods.

### 3.3 *sbgi‐ems1* plants produce more nodes

**FIGURE 3:**
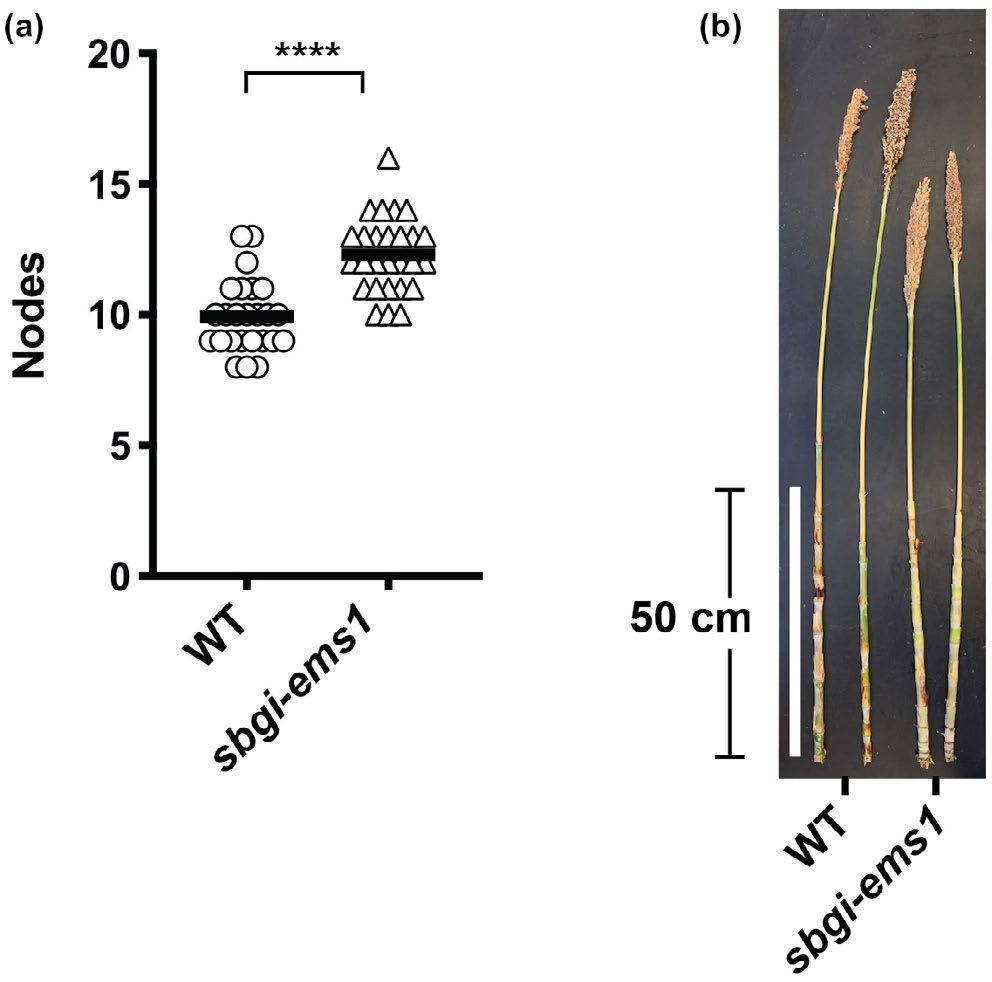
*sbgi‐ems1* mutants produce more nodes prior to flowering. (a) Number of nodes above prop roots produced by WT (circles) and *sbgi‐ems1* (triangles) BC1F3 plants from flowering time experiments. (b) Representative main stems after leaf removal of WT and *sbgi‐ems1* plants from flowering time experiments under greenhouse conditions. White bar indicates 50 cm. Statistical significance is indicated according to a two‐tailed unpaired *t*‐tests with Welch's correction at *p* < .0001 (****)

*sbgi‐ems1* plants grown under LD photoperiods produced more nodes than their WT siblings. Counting nodes for the BC1F3 plants from the greenhouse flowering trials revealed that *sbgi‐ems1* mutant plants made an average of 2.4 more than WT sibling plants (Figure 3a). While *sbgi‐ems1* mutants made additional nodes, the length of the main stem of *sbgi‐ems1* plants remained at or below that attained by WT plants (Figure 3b). These observations are consistent with additional vegetative growth prior to initiation of flowering in *sbgi‐ems1* plants, as well as more limited growth of the main stem in mutant plants after the floral transition.

### 3.4 *sbgi‐ems1* reduces expression of key flowering time genes under LD and SD photoperiods

**FIGURE 4:**
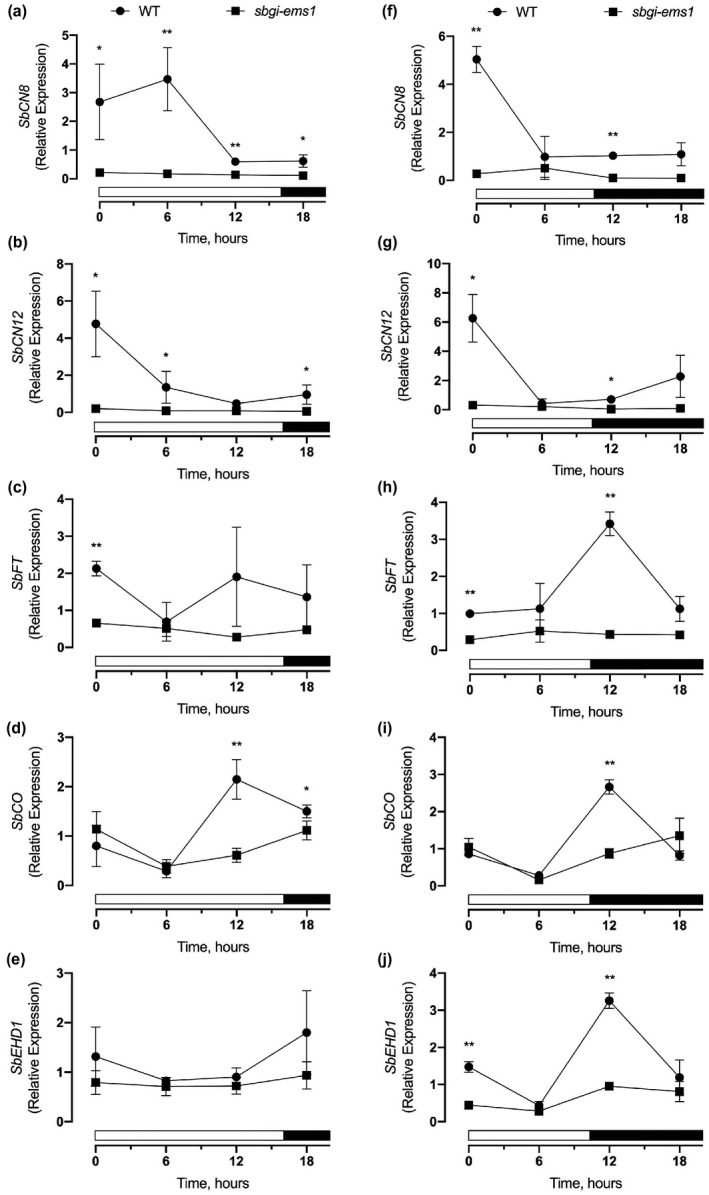
*sbgi‐ems1* alters flowering time gene expression patterns and levels. (a‐j) Relative expression levels for *SbCN8* (a, f), *SbCN12* (b, g), *SbFT* (c, h), *SbCO* (d, i), and *SbEHD1* (e, j) in leaves of WT (circles) and *sbgi‐ems1* (squares) plants grown under LD (a‐d) or SD (f‐j) conditions. X‐axis is the number of hours after dawn, white and black bars indicate light and dark periods, respectively. Time points are the average of three biological replicates for LD and two replicates for SD, and error bars are the standard deviation. Statistical significance is indicated according to the two‐tailed Student's *t*‐test at *p* < .05 (*) or < .01 (**)

To understand molecular changes associated with delayed flowering in *sbgi‐ems1*, mutant plants were tested for alterations in expression of flowering‐related genes relative to WT plants. Both LD and SD (10 hr light; 14 hr dark) photoperiods were tested to detect whether the mutant had photoperiod‐associated effects on gene expression. Leaves of WT and *sbgi‐ems1* plants at the sixth to seventh leaf stage were sampled at 0, 6, 12 and 18 hr after dawn and transcript levels assessed by qPCR. The most notable impact of the mutant allele was reduced expression of florigen‐related genes *SbCN8* and *SbCN12*. In WT plants, *SbCN8* and *SbCN12* transcripts were maximally expressed at dawn and then expression declined to basal levels by 6 to 12 hr into the day, depending on the photoperiod (Figure 4a,b,f,g). In contrast, *SbCN8* and *SbCN12* transcripts were at basal levels at all time points in *sbgi‐ems1* plants, with the greatest fold reduction at dawn. The temporal pattern of *SbFT* expression was more varied in WT, but transcript levels in *sbgi‐ems1* plants were reduced at dawn under both LD and SD conditions and at 12 hr after dawn under SD conditions (Figure 4c,h). These observations show that *SbGI* is required for expression of these three florigen‐related genes in LD and SD photoperiods.

Since the upstream action of *SbCO* and *SbEHD1* controls expression of *SbCN8*, *SbCN12*, and *SbFT* (Yang et al., 2014), we evaluated *SbCO* and *SbEHD1* expression in WT and mutant plants. *SbCO* transcript was present throughout the day in WT plants, with levels reaching their peak 12 hr after dawn under both LD and SD (Figure 4d,i). The major effect of *sbgi‐ems1* was diminishment of *SbCO* transcript levels at 12 hr after dawn in either photoperiod, a time that coincides with *SbGI* expression (Figure 1b). *SbEHD1* expression was more clearly responsive to photoperiod in WT plants: *SbEHD1* levels were largely similar across all time points under LD (Figure 4e), while a distinct peak of *SbEHD1* expression occurred at 12 hr after dawn under SD (Figure 4j). *SbEHD1* transcript levels in *sbgi‐ems1* under LD were largely similar to WT plants at all time points (Figure 4e). On the other hand, the peak of *SbEHD1* occurring at 12 hr under SD was significantly reduced in *sbgi‐ems1* (Figure 4j). These results indicate that *SbGI* promotes expression of *SbCO* under both LD and SD photoperiods, but mainly contributes to peak *SbEHD1* expression under SD.

The expression of the floral repressor *SbPRR37* was also tested to determine whether *SbGI* contributes to its regulation. In WT plants, *SbPRR37* transcript had a sharp peak of expression at 12 hr after dawn under LD and SD photoperiods, while levels in *sbgi‐ems1* plants were diminished by 2‐ to 5‐fold at these time points (Figure S3a,b). These observations show that *SbGI* activity contributes to the upregulation of *SbPRR37*.

### 3.5 SbGI interaction with SbFFL is blue light‐stimulated

**FIGURE 5:**
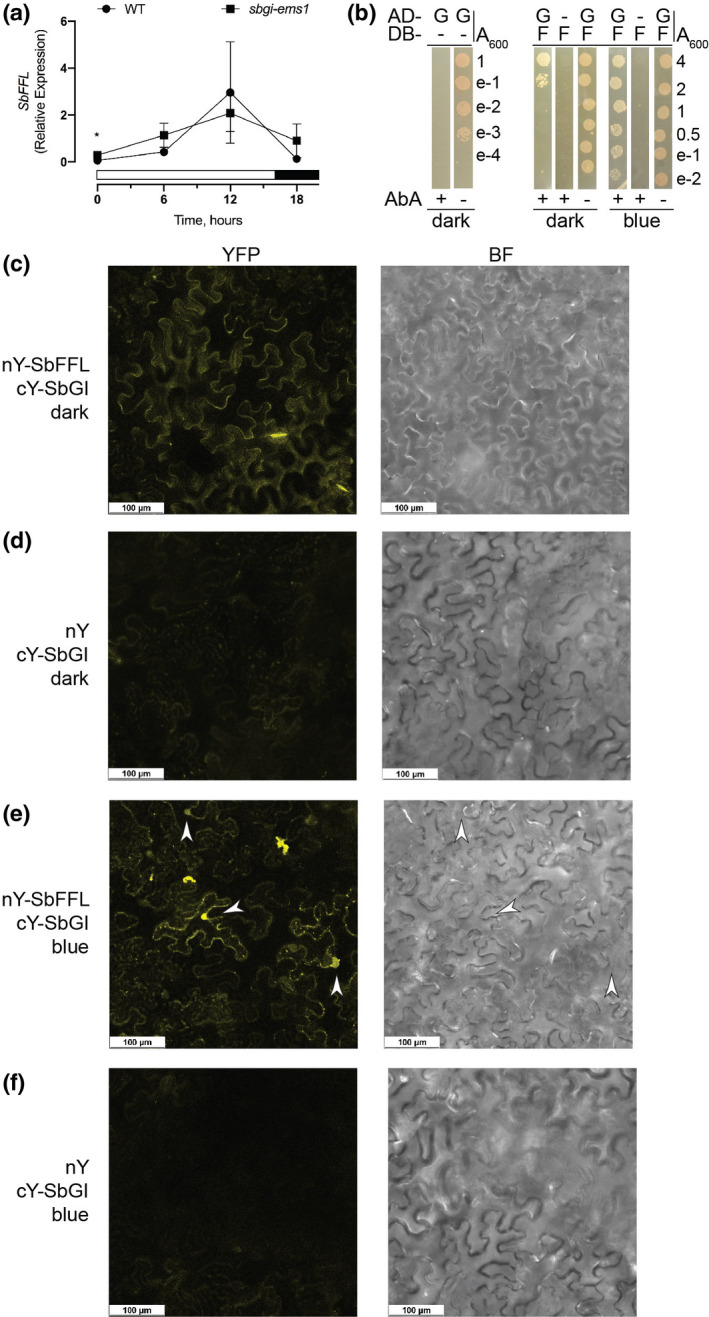
SbGI interaction with SbFFL is promoted by blue light. (a) Relative expression of *SbFFL* in WT (circles) and *sbgi‐ems1* (squares) under LD conditions. (b) Yeast two‐hybrid test for SbGI and SbFFL interaction. (c‐f) BiFC experiments with *N. benthamiana* leaves infiltrated with nY‐SbFFL and cY‐SbGI (c, e) or nY and cY‐SbGI (d, f), exposed to either continuous darkness (c, d) or blue light (e, f). For (a), X‐axis is the number of hours after dawn, white and black bars indicate light and dark periods, respectively. Time points are the average of three biological replicates and error bars are the standard deviation. For (b), yeast cultures with the combinations of AD and DB constructs, where “G” specifies SbGI, “F” specifies SbFFL, and “‐“ specifies no additional fusion, and indicated absorbance (A_600_) were spotted onto SD media lacking amino acids Leu and Trp with (+) or without (−) Aureobasidin A (AbA), providing selection for interaction, under continuous darkness (dark) or blue light (blue). Images are representative of two independent experiments. For (c‐f), fluorescence (YFP) and bright field (BF) images of *N. benthamiana* leaf sections 24–48 hr after pressure infiltration with *Agrobacterium tumefaciens* carrying the indicated nY and cY constructs and subsequent incubation under continuous darkness (dark) or blue light (blue). Arrows indicate subcellular compartments consistent with nuclei. Images are representative of fluorescent signal observed in at least 3 separate leaf sections for each of two independent experiments. Scale bar is 100 µm

Because *CO* regulation by *Arabidopsis* GI involves physical interaction between GI and FKF1, we investigated whether SbGI physically interacts with the sorghum FKF1‐like protein (Lai et al., 2020). SbFFL is over 93%–95% identical to maize orthologs FFL1 and FFL2, and 73% identical to the *Arabidopsis* FKF1 protein (Data S2). Evaluation of *SbFFL* transcript levels in leaves showed diurnal expression with a peak at 12 hr after dawn in WT plants, like *Arabidopsis* FKF1 (Nelson et al., 2000), and no significant change in expression in *sbgi‐ems1* (Figure 5a).

Tests of SbGI interaction with SbFFL by yeast two‐hybrid showed interaction between the SbGI fusion with the GAL4 activation domain (AD‐GI) and the SbFFL fusion with the GAL4 DNA‐binding domain (DB‐FFL), but not between the DB‐FFL protein and the AD alone or AD‐SbGI and the DB alone (Figure 5b). Growth of cells expressing both DB‐FFL and AD‐GI was restricted to the most concentrated yeast cultures when these were grown in the dark (A_600_ = 4 and 2; Figure 5b).

To confirm the SbGI‐SbFFL interaction, we tested whether it was enhanced by blue light since the *Arabidopsis* GI‐FKF1 interaction has this characteristic (Sawa et al., 2007). Illumination of culture plates with blue light (20 μmol/m^2^ s) during yeast growth promoted the growth of cultures carrying DB‐FFL bait and AD‐GI prey, but not those with DB‐FFL and AD alone (Figure 5b). Blue light enhancement was apparent as heavy growth for all cultures over a 400‐fold dilution series (A_600_ = 4 to 0.01; Figure 5b). These results showed that blue light promotes the SbGI‐SbFFL interaction, an expected characteristic of a bona fide SbGI interaction with SbFFL.

We sought independent validation of the SbGI‐SbFFL interaction by use of bimolecular fluorescence complementation (BiFC). This method is based on *ex vivo* reconstitution of YFP activity through interaction of one protein carrying an N‐terminal portion of YFP (nY) with another carrying a C‐terminal portion of YFP (cY; Walter et al., 2004). nY‐SbGI and cY‐SbGI were employed as a positive control for BiFC, since *Arabidopsis* GI assembles into homotetramers (Black et al., 2011) and the maize GI proteins interact with themselves and with one another (Bendix et al., 2015). As expected, transient co‐expression of nY‐SbGI and cY‐SbGI in *N. benthamiana* leaves produced strong fluorescent signal in pavement cells and a subcellular compartment likely to be the nucleus (Figure S4a), while the same pattern was not apparent when cY‐SbGI was co‐expressed with nY alone (Figure 5d). Co‐expression of nY‐SbFFL and cY‐SbGI produced fluorescent signal in the cytoplasm of pavement cells (Figure 5c). Fluorescence of comparable intensity or pattern did not occur with co‐expression of either nY or cYFP‐SbGI (Figure 5d) or nYFP‐FFL and cY (Figure S4b). These observations indicate a physical interaction between SbGI and SbFFL when expressed together in *N. benthamiana* leaves.

We investigated whether blue light altered the intensity and/or pattern of fluorescence from the nY‐SbFFL‐cY‐SbGI complex given the positive effect blue light had on the SbGI‐SbFFL interaction in the yeast two‐hybrid tests. Interestingly, exposure of infiltrated *N. benthamiana* leaves to continuous blue light enhanced fluorescent signal when cY‐GI was co‐expressed with nY‐SbFFL (Figure 5e). Blue light treatment also resulted in fluorescence signal from subcellular compartments consistent with nuclei, similar to that seen with the nY‐SbGI and cY‐SbGI combination (Figure 5e, Figure S4a). Thus, blue light may promote SbGI‐SbFFL interaction in the nucleus. Fluorescence of comparable intensity did not appear in blue light exposed leaves where cY‐SbGI and nY or nY‐SbFFL and cY were expressed together (Figure 5f, Figure S4c). These results indicate that SbGI physically interacts with SbFFL and this interaction is stimulated by blue light. In addition, the SbGI‐SbFFL complex is potentially more likely to be located in the nucleus under blue light than under dark conditions.

## 4 DISCUSSION

Identification of an EMS‐derived mutation in the *SbGI* gene, *sbgi‐ems1*, allowed for evaluation of the contribution of *SbGI* to sorghum flowering time. The *sbgi‐ems1* allele is a premature stop codon that truncates GI protein to a third of its usual length (Figure 1a). Plants homozygous for the *sbgi‐ems1* allele flower later than WT siblings under LD and SD conditions (Figure 2, Figure S2), which follows a time of extended vegetative growth indicated by additional node production (Figure 3). The delay in flowering is accompanied by a reduction in expression of genes that activate flowering, including the florigen‐related genes *SbCN8*, *SbCN12,* and *SbFT*, as well as their upstream regulators *SbCO* and *SbEHD1*. These observations provide insight into where SbGI activity fits into the regulatory networks that determine flowering time in sorghum.

The flowering behavior of *sbgi‐ems1* mutant plants indicates that *SbGI* acts early in control of flowering time. Delayed flowering time in *sbgi‐ems1* was primarily apparent as slowed time to reach boot stage, meaning the *sbgi‐ems1* are delayed in physiological processes leading up to boot stage (Figure 2; Figure S2). An early positive role *SbGI* in flowering time is consistent with the observation that *SbGI* is necessary for the proper up‐regulation of florigen‐related genes *SbCN8*, *SbCN12*, and *SbFT*. *SbCN8* and *SbCN*12 expression in WT plants had a diurnal rhythm with peak levels generally occurring at dawn, but expression of these genes in *sbgi‐ems1* plants was significantly lower at each time point and overall lacked a diurnal rhythm (Figure 4a,f,b,g). Similarly, low and arrhythmic *SbFT* expression occurred in mutant plants under SD photoperiods.

A well‐established role of florigen in multiple plant species is to promote the vegetative to floral transition at the SAM (Jaeger & Wigge, 2007; Lifschitz et al., 2006; Lin et al., 2007; Notaguchi et al., 2008; Tamaki et al., 2007). An additional role of florigen observed in wheat is triggering accumulation of growth‐promoting gibberellins (GA) in the SAM to drive spike development and shoot growth (Pearce et al., 2013). Taking into account the phenotypes associated with delayed flowering in *sbgi‐ems1* ‐ increased DTB together with greater node production and limited additional main stem growth ‐ we propose that diminished florigen expression in *sbgi‐ems1* may both delay the vegetative to reproductive transition and cause slower stem growth and panicle development after this transition.

*SbGI* appears to promote florigen‐related gene expression through up‐regulation of *SbCO* expression and, to a lesser degree and possibly only under SD, *SbEHD1* expression. Prior work indicates that *SbCO* activates *SbEHD1* expression under all photoperiod conditions and both *SbCO* and *SbEHD1* stimulate florigen gene expression (Murphy et al., 2014; Yang et al., 2014). Reduction of afternoon *SbCO* expression in the *sbgi‐ems1* background is a potential indication that *SbGI* is directly or indirectly involved in activation of *SbCO* at the transcriptional level. Also, peak *SbEHD1* expression under SD is reduced in *sbgi‐ems1*, which could arise from diminished *SbCO* or the absence of direct *SbGI* up‐regulation of *SbEHD1*.

*SbPRR37* expression is diminished in *sbgi‐ems1*, suggesting a direct or indirect regulatory role for *SbGI* in regulation of this floral repressor. Despite this observation, it is unlikely that the flowering time delay in *sbgi‐ems1* is a consequence of altered SbPRR37 protein‐directed repression of *SbEHD1* and *SbCO*. The BTx623/ATx623 genetic background used here carries the *sbprr37‐3* allele of *ma1* that encodes an inactive PRR37 protein due to a Lys162Asp substitution (Murphy et al., 2011). We also conclude that the contribution of *SbGI* to flowering time is independent of *SbPRR37* activity.

Comparing the observations for *sbgi‐ems1* to previous work on maize *gi1* mutants highlights differences in the flowering time networks in these closely related C4 grasses. The sorghum *sbgi‐ems1* allele and maize *gi1* mutants change flowering time in opposite directions. While the sorghum *sbgi‐ems1* mutant caused significantly later flowering under LD conditions, flowering time is modestly accelerated in maize *gi1* mutants under LD photoperiods (Bendix et al., 2013). *sbgi‐ems1* shows reduced expression of florigen‐related genes, indicating *SbGI* is needed for activation of *SbCO*, while upregulation of *CONZ1* and *ZCN8* is apparent in *gi1* mutant backgrounds, indicating that *GI1* is involved in *CONZ1* repression. The apparent functional dichotomy between *SbGI* and *GI1* likely reflects a dependence on underlying regulatory network architecture instead of fundamental differences in protein activity. Indeed, GI1 expressed in *Arabidopsis thaliana* complements the extreme late flowering phenotype of a *gi* knockout mutant to the same degree as *Arabidopsis* GI (Bendix, 2015), indicating that GI1 shares an inherent activity similar to *Arabidopsis* GI.

SbGI and SbFFL proteins physically interact and this interaction is stimulated by blue light (Figure 5). The SbGI‐SbFFL complex is localized in the cytosol under dark conditions, whereas in continuous blue light the protein complex appears in both the cytoplasm and a compartment consistent with the nucleus. This blue light responsive protein–protein interaction must be intrinsic to these proteins, with SbFFL likely serving as the photoreceptor, since this activity is apparent in yeast in the absence of other plant proteins. The *Arabidopsis* GI interactions with FKF1 and ZTL are blue light‐dependent (Kim et al., 2007; Krahmer et al., 2018; Pudasaini et al., 2017; Sawa et al., 2007). The FKF1‐GI complex occurs in the cytosol and the nucleus, while the ZTL‐GI interaction is proposed to be exclusively in the cytosol (Kim et al., 2007; Park et al., 2013). Like the activity of its *Arabidopsis* counterparts (Sawa et al., 2007), we predict that the SbGI interaction with SbFFL facilitates SbFKF1‐promoted relief of *SbCO* transcriptional repression and influences FKF1‐promoted stabilization of SbCO protein (Song et al., 2012, 2014).

Overall, we demonstrate that SbGI contributes to the regulatory networks controlling sorghum flowering time in a role conserved with orthologs like those from Arabidopsis, maize, and rice. In this capacity, SbGI serves as a key upstream activator of genes promoting flowering under both LD and SD photoperiods. SbGI activity is independent of the major floral repressor PRR37 but may contribute to its expression. At least one important interacting partner of SbGI is SbFFL, which perceives blue light and responds by binding to SbGI.

## CONFLICTS OF INTEREST

The authors declare no conflicts of interest.

## AUTHORS CONTRIBUTIONS

X.J. and J.C. generated and sequenced the EMS‐mutagenized sorghum population containing the *sbgi‐ems1* allele. J.C. performed early experiments on populations with the *sbgi‐ems1* allele. F.G.H conceived and designed the research. S.M.A.A. and F.G.H performed the research. S.M.A.A. and F.G.H wrote the manuscript with input from the co‐authors.

## Supporting information

Supplementary Material
