# Supplementary material for: A sorghum gigantea mutant attenuates florigen gene expression and delays flowering time
Source: Plant Direct. 2020 Nov 13;4(11):e00281. doi: 10.1002/pld3.281 (PMC7665845; doi:10.1002/pld3.281)
Supplement: Supplementary file 1 — Supplementary Material [file PLD3-4-e00281-s001.docx]

**
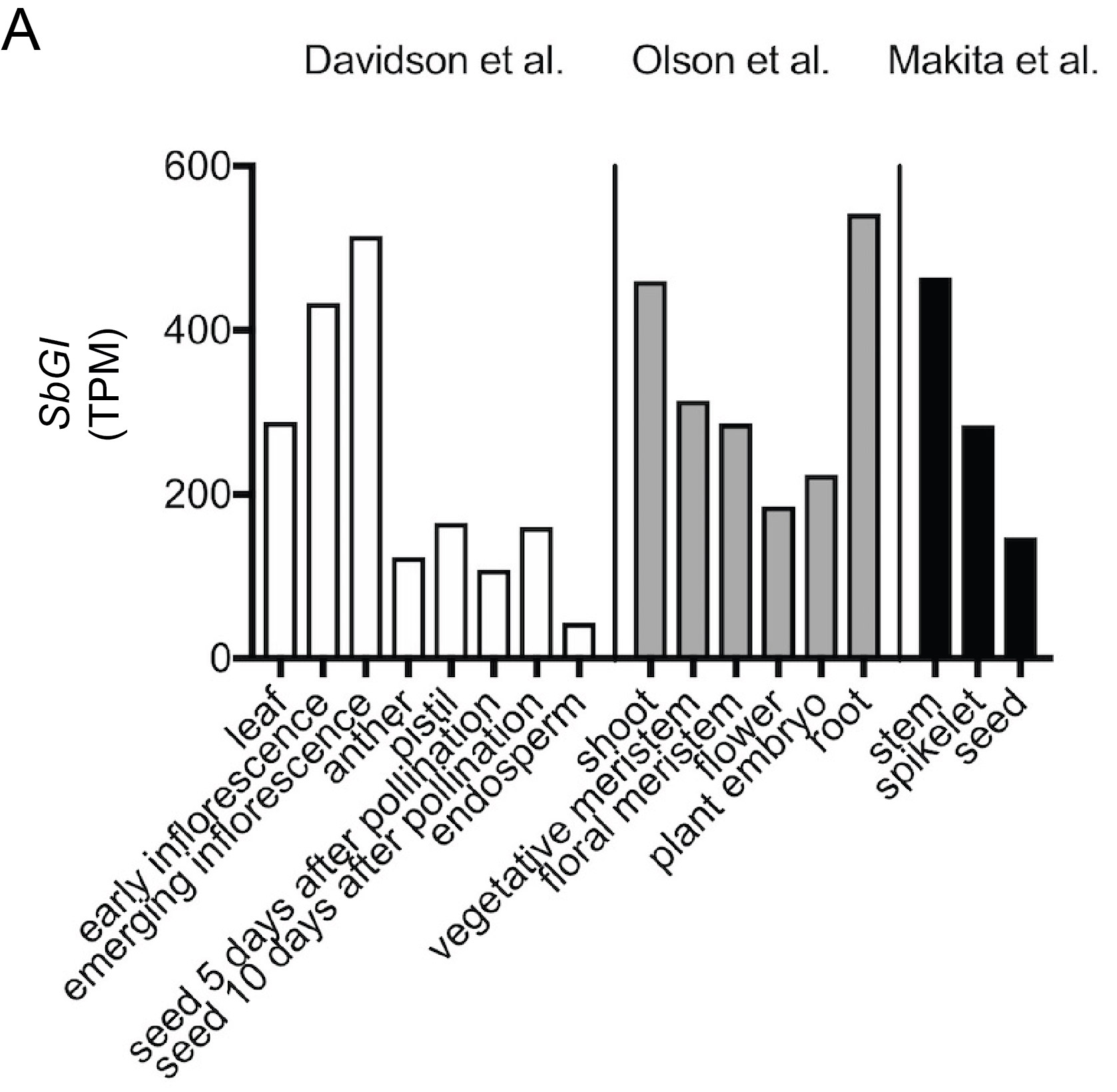
**

**Figure S1.** ***SbGI* expression in various sorghum tissues.** A) *SbGI* transcript levels in the indicated tissues according to publicly available sorghum RNA-seq datasets. TPM is transcripts per million (Li and Dewey, 2011). Data accessed at the European Molecular Biology Laboratory-European Bioinformatics Institute Expression Atlas (<https://www.ebi.ac.uk/gxa/home>) (Papatheodorou *et al.*, 2018). Specific studies are Davidson et al. 2012 (Davidson *et al.*, 2012), Olson et al. 2014 (Olson *et al.*, 2014), and Makita et al. 2015 (Makita *et al.*, 2015).


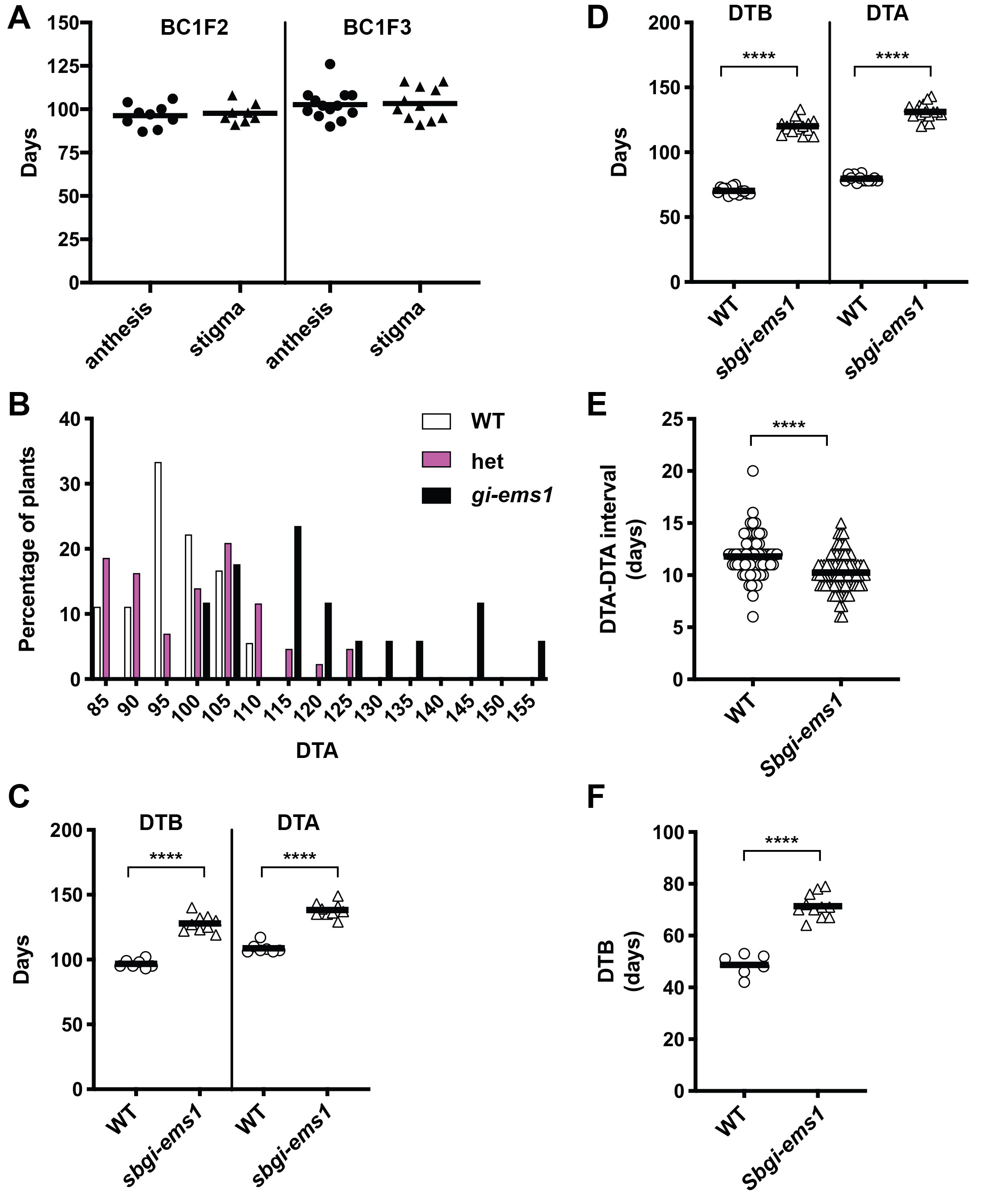


**Figure S2. Late flowering of *sbgi-ems1* plants co-segregates with the mutant allele and arises from delayed boot stage.** A) DTA (anthesis) or days to sigma exertion (stigma) for BC1F2 and BC1F3 WT plants. B) DTA for WT (white bars), heterozygous (het; magenta bars), and *sbgi-ems1* (black bars*)* plants from three flowering trials with the BC1F2 population under LD greenhouse conditions. C) and D) DTA and DTB for *sbgi-ems1* and WT BC2F3 (C) and BC4F3 (D) individuals under LD greenhouse conditions. E) The days between DTB and DTA (DTA-DTB interval) for BC1F3 *sbgi-ems1* and WT sibling individuals in LD greenhouse flowering time trials. F) DTB for BC1F3 *sbgi-ems1* and WT siblings grown in the field in Davis, CA during summer of 2019. C-F) All measurements are shown for *sbgi-ems1* (triangles) and WT (circles), and bar indicates the average of all measurements. Statistical significance is indicated according to a two-tailed unpaired t-tests with Welch’s correction at p-value < 0.0001 (****), < 0.001 (***), < 0.01 (**) and < 0.05 (*).


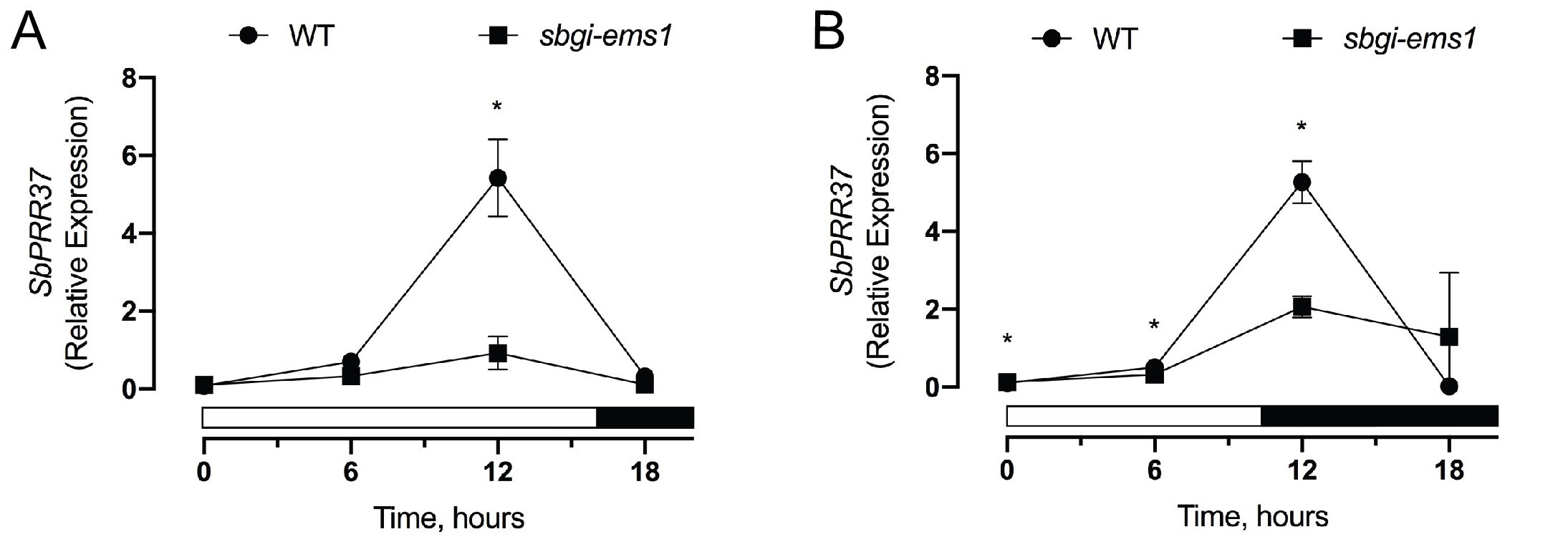


**Figure S3. Expression of floral repressor *SbPRR37* in *sbgi-ems1* and WT plants under LD and SD.** Relative expression of *SbPRR37* in leaves of *sbgi-ems1* (squares) BC1F3 plants and WT siblings (circles) siblings at 6^th^ leaf stage grown under LD (A) and SD (B) conditions. X-axis is the number of hours after dawn, white and black bars indicate light and dark periods, respectively. Time points are the average of two biological replicates and error bars are the standard deviation. Statistical significance is indicated according to the two-tailed Student’s t-test at p-value < 0.05 (*).


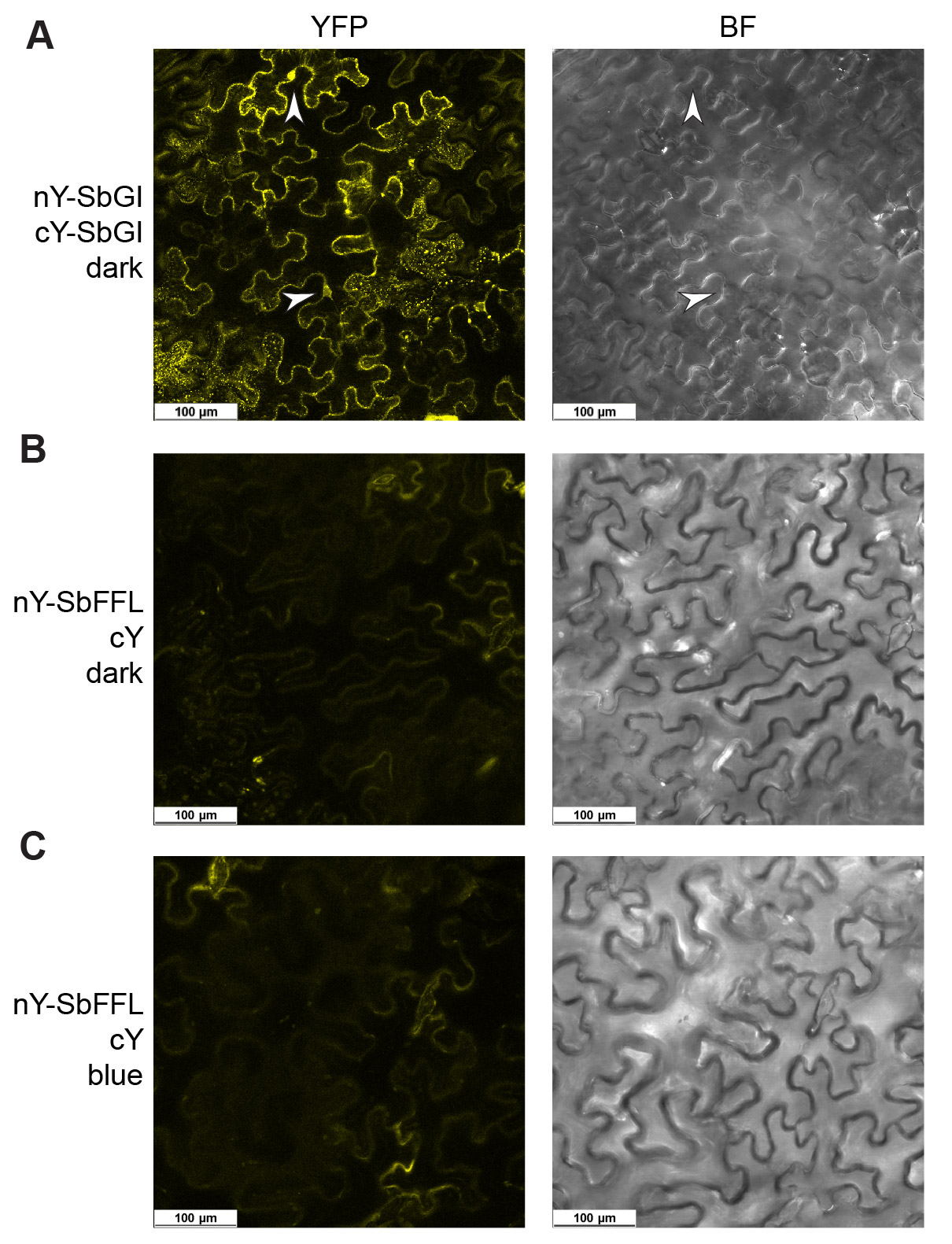


**Figure S4. BiFC results for the combinations of nY-SbGI/cY-SbGI and nY-SbFFL/cY.** BiFC experiments with *N. benthamiana* leaves infiltrated with nY-SbGI and cY-SbGI (A) or nY-FFL and cY (B, C) exposed to either continuous darkness (A, B) or blue light (C). Fluorescence (YFP) and bright field (BF) images of *N. benthamiana* leaf sections 24-48 hours after pressure infiltration with *Agrobacterium tumefaciens* carrying the indicated nY and cY constructs and subsequent incubation under continuous darkness (dark) or blue light (blue). Arrows indicate subcellular compartments consistent with nuclei. Images are representative of fluorescent signal observed in at least 3 separate leaf sections for each of two independent experiments. Scale bar is 100 µm.

**Data S1. Amino acid alignment of GI proteins from sorghum, maize, and *Arabidopsis*.**

Percent Identity Matrix - created by Clustal2.1

1: GI_Sbicolor 100.00 96.47 96.21 68.20

2: GI1_Zmays 96.47 100.00 95.00 68.54

3: GI2_Zmays 96.21 95.00 100.00 68.49

4: GI_A_thaliana 68.20 68.54 68.49 100.00

GI_S_bicolor --MSDSNVKWIDGLQFTSLYWPPPQDVEQKQAQILAYVEYFGQFTADSEQFPEDVAQLIQ 58

GI1_Z_mays --MSDSNVKWIDGLQFTSLYWPPPLDAEQKQAQILAYVEYFGQFTADTDQFPEDIAQLIQ 58

GI2_Z_mays --MSESNVKWIDGLHFTSLYWPPPQDVEQKQAQILAYVEYFGQFTADSEQFPEDVAQLIQ 58

GI_A_thaliana MASSSSSERWIDGLQFSSLLWPPPRDPQQHKDQVVAYVEYFGQFT--SEQFPDDIAELVR 58

*.*. :*****:*:** **** * :*:: *::********** ::***:*:*:*::

GI_S_bicolor SSYPSKESRLVDEVLATFVLHHPEHGHAVVHPILSRIIDGTLCYDRHGPPFSSFISLFSH 118

GI1_Z_mays SSYPSKENRLVDEVLATFVLHHPEHGHAVAHPILSRIIDGTLCYDRHGPPFSSFISLFSH 118

GI2_Z_mays SSYPSKESRLIDEVLATFVLHHPEHGHAVVHPILSPIIDGTLCYDRHGPPFSSFISLFSH 118

GI_A_thaliana HQYPSTEKRLLDDVLAMFVLHHPEHGHAVILPIISCLIDGSLVYSKEAHPFASFISLVCP 118

.***.*.**:*:*** ************ **:* :***:* *.:.. **:*****..

GI_S_bicolor TSEQEYSEQWALACGEILRVLTHYNRPIFKVERQHSEAECSSTSDQATSSDSTDKKSNNS 178

GI1_Z_mays NSEQEYSEQWALACGEILRVLTHYNRPIFKVERQHTEAECSSTSDQATSSDSTDKRSNNS 178

GI2_Z_mays TSEQEYSEQWALACGEILRVLTHYNRPIFKVERQHSEAECSTTSDQATSSDSTDKKSNNS 178

GI_A_thaliana SSENDYSEQWALACGEILRILTHYNRPIYKTEQQNGDTERNCLSKATTSGSPT-SEPKAG 177

.**::**************:********:*.*:*: ::* . *. :**.. * .. : .

GI_S_bicolor PGNESDRKPLRPLTPWITDILLAAPLGIRSDYFRWCGGVMGKYAAGGELKPPTTACSRGS 238

GI1_Z_mays PGNESDWKPLRPLTPWITDILLAAPLGIRSDYFRWCGGVMGKYAAGGELKPPTTACSRGS 238

GI2_Z_mays LGNESDRKPLRPLTPWITDILLAAPLGIRSDYFRWCSGVMGKYAAGGELKPPTTAYSRGS 238

GI_A_thaliana SPTQHERKPLRPLSPWISDILLAAPLGIRSDYFRWCSGVMGKYAAG-ELKPPT-IASRGS 235

.: : ******:***:******************.********* ****** ****

GI_S_bicolor GKHPQLMPSTPRWAVANGAGVILSVCDEEVARYETANLTAAAVPALLLPPPTTPLDEHLV 298

GI1_Z_mays GKHPQLMPSTPRWAVANGAGVILSVCDEEVARYETANLTAAAVPALLLPPPTTPLDEHLV 298

GI2_Z_mays GKHPQLMPSTPRWAVANGAGVILSVCDEEVARYETANLTAAAVPALLLPPPTTPLDEHLV 298

GI_A_thaliana GKHPQLMPSTPRWAVANGAGVILSVCDDEVARYETATLTAVAVPALLLPPPTTSLDEHLV 295

***************************:********.***.************ ******

GI_S_bicolor AGLPPLEPYARLFHRYYAIATPSATQRLLFGLLEAPPSWAPDALDAAVQLVELLRAAEDY 358

GI1_Z_mays AGLPPLEPYARLFHRYYAIATPSATQRLLFGLLEAPPSWAPDALDAAVQLVELLRAAEDY 358

GI2_Z_mays AGLPPLEPYARLFHRYYAIATPSATQRLLFGLLEAPPSWAPDALDAAVQLVELLRAAEDY 358

GI_A_thaliana AGLPALEPYARLFHRYYAIATPSATQRLLLGLLEAPPSWAPDALDAAVQLVELLRAAEDY 355

**** ************************:******************************

GI_S_bicolor ASGMRLPKNWMHLHFLRAIGTAMSMRAGIAADTAAALLFRILSQPTLLFPPLRHAEGVEV 418

GI1_Z_mays ASGMRLPKNWMHLHFLRAIGTAMSMRAGIAADTAAALLFRILSQPTLLFPPLRHAEGVEV 418

GI2_Z_mays ASGMRLPKNWMHLHFLRAIGTAMSMRAGIAADTAAALLFRILSQPTLLFPPLRHAEGVDV 418

GI_A_thaliana ASGVRLPRNWMHLHFLRAIGIAMSMRAGVAADAAAALLFRILSQPALLFPPLSQVEGVEI 415

***:***:************ *******:***:************:****** :.***::

**|*Sbgi-ems1* - W463***

GI_S_bicolor HHEPLGGYVSSYKKQLEVPASEATIDATAQGIASLLCAHGPDVE**W**RICTIWEAAYGLLPL 478

GI1_Z_mays HHEPLGGYVSSYKKQLEVPASEATIDATAQGIASLLCAHGPDVEWRICTIWEAAYGLLPL 478

GI2_Z_mays HHEPLGGYVSSYKKQLEVPASEATIDATAQGIASLLCAHGPDVEWRICTIWEAAYGLLPL 478

GI_A_thaliana QHAPIGGYSSNYRKQIEVPAAEATIEATAQGIASMLCAHGPEVEWRICTIWEAAYGLIPL 475

:* *:*** *.*:**:****:****:********:******:***************:**

GI_S_bicolor SSSAVDLPEIVVAAPLQPPTLSWSLYLPLLKVFEYLPRGSPSEACLMRIFVATVEAILRR 538

GI1_Z_mays SSSAVDLPEIVVAAPLQPPTLSWSLYLPLLKVFEYLPRGSPSEACLMRIFVATVEAILRR 538

GI2_Z_mays SSSAVDLPEIVVAAPLQPPTLSWNLYLPLLKVFEYLPRGSPSEACLMRIFVATVEAILRR 538

GI_A_thaliana NSSAVDLPEIIVATPLQPPILSWNLYIPLLKVLEYLPRGSPSEACLMKIFVATVETILSR 535

.*********:**:***** ***.**:*****:**************:*******:** *

GI_S_bicolor AFPSETSEQS-------RKPRSQSKNLAVAELHTMIHSLFVESCASMDLASRLLFVVLTV 591

GI1_Z_mays TFPSETSEQS-------RKPRSQSKNLAVAELHTMIHSLFVESCASMDLASRLLFVVLTV 591

GI2_Z_mays AFPSETPEQS-------RKPRSQSKNLAVAELHTMIHSLFVESCASMDLASRLLFVVLTV 591

GI_A_thaliana TFPPESSRELTRKARSSFTTRSATKNLAMSELRAMVHALFLESCAGVELASRLLFVVLTV 595

:** *: .: . ** :****::**::*:*:**:****.::************

CLUSTAL O(1.2.4) multiple sequence alignment

An * (asterisk) indicates positions which have a single, fully conserved residue.
A : (colon) indicates conservation between groups of strongly similar properties - scoring > 0.5 in the Gonnet PAM 250 matrix.
A . (period) indicates conservation between groups of weakly similar properties - scoring =< 0.5 in the Gonnet PAM 250 matrix.

**Data S2. Amino acid alignment of FKF1 protein from *Arabidopsis* and FFL proteins from sorghum and maize.**

Percent Identity Matrix - created by Clustal2.1

1: SbFFL_S_bicolor 100.00 95.23 93.88 73.24

2: ZmFFL1_Z_mays 95.23 100.00 92.47 71.88

3: ZmFFL2_Z_mays 93.88 92.47 100.00 72.62

4: FKF1_A_thaliana 73.24 71.88 72.62 100.00

SbFFL_S_bicolor --------------------------------MEVDAE----AGWPQWGAPAA**AAAAGLG** 24

ZmFFL1_Z_mays -----MFDDAGAVAVKRMRLWEEDEVEVEEEGMEVDAE----PGWP-WGTPAA------G 44

ZmFFL2_Z_mays -----------------MRLWEEEDDDEDEEGMEVDGEAEDEPGWP-----CGAPEAGPG 38

FKF1_A_thaliana MAREHAIGEATGKRKKRGRV--EEAEEYCNDGIEEQVEDEKLPLE--VG-MF------YY 49

:* : *

**PAS domain**

SbFFL_S_bicolor **EPRAAAIVVADASEVDFPVIYVNAAFEAATGYRAHEVLGRNCRFLQFRDPRAQRRHPLVD** 84

ZmFFL1_Z_mays LSRAAAIVVADAAEPDFPVIYVNAAFESATGYRAHEVLGRNCRFLQFRDPRAQRRHPLVD 104

ZmFFL2_Z_mays ETRPAAILVADAAEVDFPVIYVNAAFEAATGYRAHEVLGRNCRFLQFRDPHAQRRHPLVD 98

FKF1_A_thaliana PMTPPSFIVSDALEPDFPLIYVNRVFEVFTGYRADEVLGRNCRFLQYRDPRAQRRHPLVD 109

:::*:** * ***:**** .** *****.***********:***:*********

**PAC domain**

SbFFL_S_bicolor **PMV**VSEIRRCLNEGIEF**QGELLNFRKDGAPLYNRLRLIPMHGDDGYVTHVIGIQLFSDA**N 144

ZmFFL1_Z_mays PMVVSEIRRCLNEGIEFHGELLNFRKDGAPLYNRLSLIPMHGDDGYVTHVIGIQLFSEAN 164

ZmFFL2_Z_mays PMVVSEIRRCLSEGIEFQGELLNFRKDGAPLHNRLRLVPMHGDDGYVTHVIGIQLFSEAN 158

FKF1_A_thaliana PVVVSEIRRCLEEGIEFQGELLNFRKDGTPLVNRLRLAPIRDDDGTITHVIGIQVFSETT 169

*:*********.*****:**********:** *** * *::.*** :*******:**::.

SbFFL_S_bicolor IDLSSVSYPVYKQQS-NRLSIQDLNSASHEHAPKIQSSDHC**AIFQLSDEVLAHNILSRLS** 203

ZmFFL1_Z_mays IDLSSVSYPVYKQQTNNRPSIQDLNSASHEHAPKVQSADHCGILQLSDEVLAHNILSRLS 224

ZmFFL2_Z_mays IDLSSVSYPVYKQKSSSRPSIQDLNSSPHEHAPKIQSADHCGMLQLSDEVLAHNILSRLS 218

FKF1_A_thaliana IDLDRVSYPVFKHKQQLDQTSECLFPS-GSPRFKEHHEDFCGILQLSDEVLAHNILSRLT 228

***. *****:*:: : : * : . * : *.*.::***************:

**F-box domain**

SbFFL_S_bicolor **PRDVASIGSVCTRMHELTKNNHLRKMVCQ**NAWGRDVTVRLEMSTKMVGWGRLARELTTLE 263

ZmFFL1_Z_mays PRDVASIGSVCTRMHELTKNDHLRKMVCQNAWGRDATVKLEMSTKMVGWGRLARELTTLE 284

ZmFFL2_Z_mays PRDVASIGSVCTRMHELTKNDHLRKMVCQNAWGRDVTVRLEMSTKMVGWGRLARELTTLE 278

FKF1_A_thaliana PRDVASIGSACRRLRQLTKNESVRKMVCQNAWGKEITGTLEIMTKKLRWGRLARELTTLE 288

*********.* *:::****: :**********:: * **: ** : ************

**Kelch_4 domain**

SbFFL_S_bicolor AASWRKFTVGGRVEP**SRCNFSACAVGNRLVLFGGEGVNMQPMDDTFVLNLEAARPEWRRV** 323

ZmFFL1_Z_mays AASWRKFTVGGRVEPSRCNFSACAVGNRLVLFGGEGVNMQPMDDTFVLNMEAARPEWRRV 344

ZmFFL2_Z_mays AASWRKFTVGGRVEPSRCNFSACAVGNRLVLFGGEGVNMQPMDDTFVLNLEAATPEWRRV 338

FKF1_A_thaliana AVCWRKFTVGGIVQPSRCNFSACAVGNRLVLFGGEGVNMQPLDDTFVLNLDAECPEWQRV 348

*..******** *:***************************:*******::* ***:**

**Kelch_4 domain**

SbFFL_S_bicolor **KVSASPPGRWGHTLSWLNGSWLVVFGGCGQQGLLNDVFVLDLDAQQPTWREVASEAPPLP** 383

ZmFFL1_Z_mays KVSASPPGRWGHTLSWLNGSWLVVFGGCGQQGLLNDVFVLDLDAQQPTWREVASEGPPLP 404

ZmFFL2_Z_mays KVSASPPGRWGHTLSWLNGSWLVVFGGCGQQGLLNDVFVLDLDAQQPTWREVASEGPPLP 398

FKF1_A_thaliana RVTSSPPGRWGHTLSCLNGSWLVVFGGCGRQGLLNDVFVLDLDAKHPTWKEVAGGTPPLP 408

:*::*********** *************:**************::***:***. ****

**Kelch_4 domain**

SbFFL_S_bicolor **RSWHSSCTLDGSKLVVSGGCTESGVLLSDTFLLDLTKEKPAWREIPTSWSPPS**RLGHTMS 443

ZmFFL1_Z_mays RSWHSSCTLDGSKLVVSGGCTESGVLLSDTFLLDLTKEKPAWREIPTSWSPPSRLGHTMS 464

ZmFFL2_Z_mays RSWHSSCTLDGSKLVVSGGCAESGVLLSDTFLLDLTKEKPAWREIPTSWSPPSRLGHTTS 458

FKF1_A_thaliana RSWHSSCTIEGSKLVVSGGCTDAGVLLSDTFLLDLTTDKPTWKEIPTSWAPPSRLGHSLS 468

********::**********:::*************.:**:*:******:*******: *

SbFFL_S_bicolor VYGTTKLFMFGGLAKSGSLRLRSSDAYSIDVSEDSPQWRQLAT-TGFPNVGPP**PRLDHVA** 502

ZmFFL1_Z_mays VYGTTKLLMFGGLAKSGSLRLRSSDAYTMDVGEDSPQWRQLAT-TGFPNVGPPPRLDHVA 523

ZmFFL2_Z_mays VYGATKLFMFGGLAKSGSLRLRSSDAYTVDVSEDSPQWRQLATTTGFPNVSPPPRLDHVA 518

FKF1_A_thaliana VFGRTKILMFGGLANSGHLKLRSGEAYTIDLEDEEPRWRELECSAFPGVVVPPPRLDHVA 528

*:* **::******:** *:***.:**::*: ::.*:**:* : * *********

**Kelch_2 domain**

SbFFL_S_bicolor **VSLPCGRIIIFGGSIAGLHSPAQLFLIDPAEEKPTWRILNVPG**QPPKFAWGHSTCVVGGT 562

ZmFFL1_Z_mays VSLPCGRIIIFGGSIAGLHSPAQLFLVDPAEEKPTWRILNVPGKPPKFAWGHSTCVVGGT 583

ZmFFL2_Z_mays VSLPCGRIIIFGGSIAGLHSPAQLFLIDPAEEKPIWRILNVPGQPPKFAWGHSTCVVGGT 578

FKF1_A_thaliana VSMPCGRVIIFGGSIAGLHSPSQLFLIDPAEEKPSWRILNVPGKPPKLAWGHSTCVVGGT 588

**:****:*************:****:******* ********:***:************

**Supporting Information References**

Davidson, R. M. *et al.* (2012) ‘Comparative transcriptomics of three Poaceae species reveals patterns of gene expression evolution.’, *The Plant Journal*, 71(3), pp. 492–502. doi: 10.1111/j.1365-313X.2012.05005.x.

Li, B. and Dewey, C. N. (2011) ‘RSEM: Accurate transcript quantification from RNA-Seq data with or without a reference genome’, *BMC Bioinformatics*, 12. doi: 10.1186/1471-2105-12-323.

Makita, Y. *et al.* (2015) ‘MOROKOSHI: transcriptome database in Sorghum bicolor.’, *Plant & Cell Physiology*, 56(1), p. e6. doi: 10.1093/pcp/pcu187.

Olson, A. *et al.* (2014) ‘Expanding and Vetting Gene Annotations through Transcriptome and Methylome Sequencing’, *The Plant Genome*. doi: 10.3835/plantgenome2013.08.0025.

Papatheodorou, I. *et al.* (2018) ‘Expression Atlas: Gene and protein expression across multiple studies and organisms’, *Nucleic Acids Research*, 46(D1), pp. D246–D251. doi: 10.1093/nar/gkx1158.
